# Using open source accelerometer analysis to assess physical activity and sedentary behaviour in overweight and obese adults

**DOI:** 10.1186/s12889-018-5215-1

**Published:** 2018-04-23

**Authors:** Paul Innerd, Rory Harrison, Morc Coulson

**Affiliations:** School of Nursing and Health Sciences, Faculty of Health Sciences and Wellbeing, University of Sunderland, Sunderland, SR1 3SD UK

**Keywords:** Physical activity, Sedentary behaviour, Measurement, Accelerometer, Questionnaire, Overweight, obese

## Abstract

**Background:**

Physical activity and sedentary behaviour are difficult to assess in overweight and obese adults. However, the use of open-source, raw accelerometer data analysis could overcome this. This study compared raw accelerometer and questionnaire-assessed moderate-to-vigorous physical activity (MVPA), walking and sedentary behaviour in normal, overweight and obese adults, and determined the effect of using different methods to categorise overweight and obesity, namely body mass index (BMI), bioelectrical impedance analysis (BIA) and waist-to-hip ratio (WHR).

**Methods:**

One hundred twenty adults, aged 24–60 years, wore a raw, tri-axial accelerometer (Actigraph GT3X+), for 3 days and completed a physical activity questionnaire (IPAQ-S). We used open-source accelerometer analyses to estimate MVPA, walking and sedentary behaviour from a single raw accelerometer signal. Accelerometer and questionnaire-assessed measures were compared in normal, overweight and obese adults categorised using BMI, BIA and WHR.

**Results:**

Relationships between accelerometer and questionnaire-assessed MVPA (Rs = 0.30 to 0.48) and walking (Rs = 0.43 to 0.58) were stronger in normal and overweight groups whilst sedentary behaviour were modest (Rs = 0.22 to 0.38) in normal, overweight and obese groups. The use of WHR resulted in stronger agreement between the questionnaire and accelerometer than BMI and BIA. Finally, accelerometer data showed stronger associations with BMI, BIA and WHR (Rs = 0.40 to 0.77) than questionnaire data (Rs = 0.24 to 0.37).

**Conclusions:**

Open-source, raw accelerometer data analysis can be used to estimate MVPA, walking and sedentary behaviour from a single acceleration signal in normal, overweight and obese adults. Our data supports the use of WHR to categorise overweight and obese adults. This evidence helps researchers obtain more accurate measures of physical activity and sedentary behaviour in overweight and obese populations.

## Background

Strategies to prevent and treat obesity typically promote increased physical activity [1] and reduced sedentary behaviour [2]. Therefore, accurate measurements of physical activity and sedentary behaviour in overweight and obese individuals are essential. At present, the measurement of physical activity and sedentary behaviour is carried out using either subjective or objective methods [3]. Many studies have used questionnaires to subjectively assess physical activity and sedentary behaviour [4, 5]. Questionnaires are inexpensive, easy to administer and allow data to be gathered about physical activity intensity (sedentary, light, moderate, vigorous) and physical activity type, such as sitting and walking, which provides useful contextualisation to the data [6]. However, the subjective nature of questionnaires often results in large measurement error [7].

Accelerometers are becoming increasingly cost-effective and technologically advanced [8]. New accelerometer models such as the ActiGraph GT3X [9], GENEActiv [10], and Axivity AX3 [11] provide access to high-resolution (≤100 Hz), raw acceleration data compatible with open-source, freely available analytical methods which estimate physical activity intensity [12], physical activity type [13] and sedentary behaviour [14]. This approach allows the user to obtain a suite of measures from one acceleration signal. Hence, population-based studies such as the National Health and Nutritional Examination Survey (NHANES) [8] the Whitehall II Study [15] and UK Biobank [11] are moving toward the use of raw accelerometer signals. However, the presence of overweight and obesity on the relationship between raw accelerometer and questionnaire-assessed physical activity is poorly understood.

Overweight and obesity is typically classified using different methods across studies, such as body mass index (BMI), bioelectrical impedance analysis (BIA) or waist circumference/waist-to-hip ratio (WHR) [16]. The widespread use of BMI, BIA and WHR is mainly due to their feasibility. However, these methods do not measure the same thing. BMI (normal, overweight, obese) has been criticised for not accurately identifying high adiposity associated with poor health [17], in one study misclassifying 25% men and 48% women as obese [18]. The accuracy of BIA (average, high, obese) is heavily influenced by body fat distribution, age and hydration levels varying by up to 10% [19] and is therefore of limited use in populations other than healthy, euvolemic adults [20]. Some studies report that measures of central adiposity such as WHR (normal, overweight, obese) are better discriminators of unhealthy body composition [21]. However, there is no consensus regarding which method is best.

Raw accelerometer and questionnaire-assessed physical activity is yet to be compared in normal, overweight and obese adults. The aims of this study were to; 1) examine whether the relationship between accelerometer and questionnaire-assessed physical activity differs in normal, overweight and obese adults regardless of the method of adiposity grouping; 2) determine whether the method of adiposity grouping (BMI, BIA or WHR) affects the relationship between questionnaire and accelerometer-assessed physical activity; 3) quantify the association between accelerometer and questionnaire-assessed physical activity intensity, activity type and sedentary behaviour with adiposity group (BMI, BIA or WHR).

## Methods

### Participants

One hundred twenty adults aged 24 to 60 years were recruited from the University of Sunderland staff and student body via a study advert distributed by email. Selection criteria were age over 18 years, not underweight, no chronic diseases such as cardiovascular disease, stroke, COPD, or reduced functional capacity. Detailed information regarding the purpose and methods used in the study was provided and a medical screening questionnaire was completed. All participants provided written informed consent. All data collection was carried out in accordance with the Declaration of Helsinki. The study protocol was approved by the University of Sunderland Ethics Committee.

### Questionnaire-assessed physical activity

Participants completed a modified version of the International Physical Activity Questionnaire-Short Form (IPAQ-S) at the end of each day. The IPAQ-S is a widely used, 7 item questionnaire in which participants are required to recall their physical activity over the past 7 days. We modified the questionnaire so that participants were asked to recall their physical activity at the end of each day not each week so that questionnaire data was directly comparable to accelerometer data. Questions were as follows: “How much time did you spend doing moderate physical activities like carrying light loads or bicycling at a regular pace?” “How much time did you spend doing vigorous physical activities like heavy lifting, digging, aerobics, or fast bicycling today?” Responses from these questions were combined to give time spent in moderate to vigorous physical activity (MVPA). Further questions were, “How much time did you spend walking today?” and “How much time did you spend sitting today? This may include time spent sitting at a desk, visiting friends, reading, or sitting or lying down to watch television, but not sleeping.” Participants also kept a sleep diary, noting the times they went to sleep, when they woke up and if they napped during the day. We then compared questionnaire and accelerometer-assessed measures of MVPA, walking and sedentary behaviour.

### Accelerometer-assessed physical activity

**Fig. 1:**
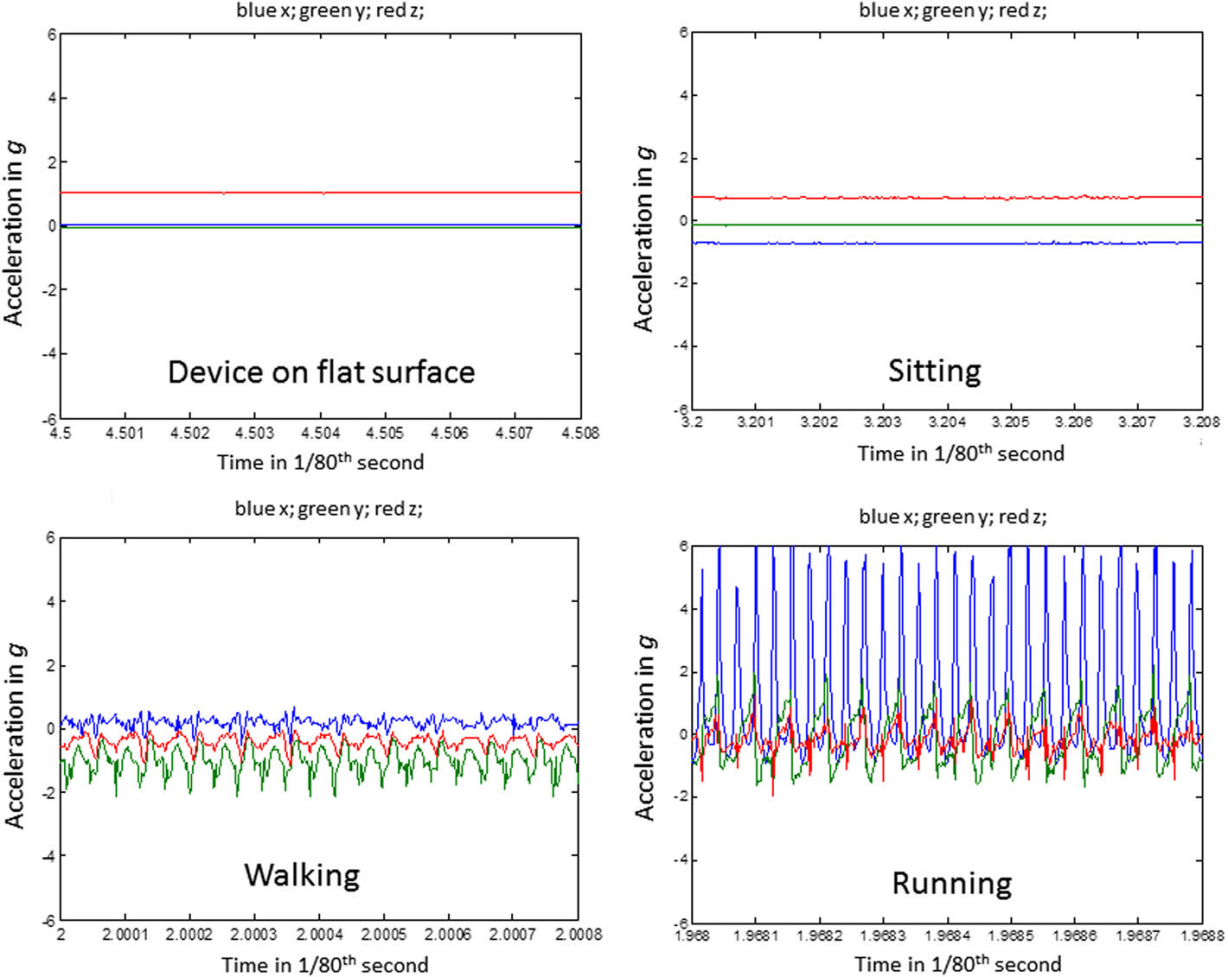
Raw tri-axial accelerometer output (*g*) showing the device flat on a surface, sitting, walking and running

Each participant wore an accelerometer (ActiGraph GT3X+, Actigraph Inc., Pensacola, FL) on their waist for three days. The device was worn continuously to capture continuous raw acceleration data needed for analysis. Three days continuous wear was considered most feasible as participants with high central adiposity can encounter discomfort from the waistband [22]. Accelerometers were initiated to start recording at 0900 h on day 1 and stop recording 0900 h on day 4. Data was sampled at 60 Hz, in keeping with other method comparison studies using hip worn raw acceleration data [10] and were stored in gravity (*g*) units (1 *g* = 9.81 m/s^2^). The vector magnitude was taken from the three axes and then subtracted by the value of gravity (*g*) as in $\sqrt{\mathrm{x}2+\mathrm{y}2+\mathrm{z}2}\hbox{-} 1$ after which, negative values were rounded up to zero, referred to as Euclidian Norm Minus One or ENMO [23]. The resulting values are expressed in milligravity (m*g*), where 1000 m*g* = 9.81 m/s^2^. The average of these values was calculated over a 1 s epoch. Signal processing was done in open-source programme R (http://cran.r-project.org/). If the reader is interested in replicating these accelerometer analyses with free-living accelerometer data, we recommend using the R-package GGIR which facilitates data cleaning, non-wear detection and the extraction of user-defined acceleration levels. To assess time spent in MVPA we used the cut-point of 70 m*g* which has accurately classified MVPA from raw acceleration data at the hip [10]. The classification of sedentary and walking activities was carried out using activity type classification which involves the detection of features in the raw acceleration signal to distinguish activities by type [24]. Figure 1 shows visualisation of the raw acceleration signal during selected activities of daily living. Readers interested in reproducing this method are referred to the following study [13]. This analysis detects sitting and supine positions but does not differentiate sedentary behaviour from sleep. Therefore, participants kept a sleep diary to record sleep duration (mins/day), which was subtracted from accelerometer sedentary time before further analysis.

### Body composition

Indicators of overweight and obesity were assessed using BMI, BIA and WHR. BMI was calculated from the participants’ weight (kg) divided by height (m^2^). Normal weight was defined as BMI ≤24.9, overweight as BMI 25–29.9 and obese as BMI ≥30 [25]. BIA was used to estimate percent body fat (Helios, Forana, Frankfurt, Germany). The following age-specific cut-offs were used for women; average 22–25%, high 26–36%, obese > 36%, and for men; average 14–20%, high 21–25%, obese > 25% based on current standards from the American College of Sports Medicine [26]. Waist and hip circumference was measured with participants standing in a relaxed position using a SECA 201 measuring tape (SECA GmbH&Co, Hamburg, Germany). WHR ratio was categorised as normal (< 0.8 for women, < 0.9 for men), overweight (0.80–0.84 for women, 0.90–0.99 for men) and obesity (> 0.85 for women, > 1.0 for men) [27].

Demographic factors including sex, smoking status, alcohol intake and educational status were recorded. Alcohol intake was categorised as less than once per week, 1 or 2 times a week and several times a week or daily. Education was assessed by taking the highest qualification attained upon leaving full-time education and was categorised as GCSE/O level, A-level, University degree and Postgraduate.

### Statistical analysis

As the data were not normally distributed, we used non-parametric Spearman’s rank correlations with 95% confidence intervals (CI; derived using Fisher’s z transformation) to show associations between sedentary time, walking and MVPA (mins/day) assessed by questionnaire and accelerometer. The analyses were stratified by BMI, BIA, WHR, smoking status, alcohol intake and education level to determine whether the relationships between accelerometer and questionnaire-derived measures were stronger in certain adiposity groups and demographic groups. Mean differences between questionnaire and accelerometer-assessed values for sedentary time, walking and MVPA were compared using paired samples t-tests and the bias and variability between the two measurement methods determined using a limits of agreement approach [28]. The associations of adiposity categories with physical activity measures (sedentary time, walking and MVPA) from the questionnaire and accelerometer were examined using Spearman’s rank correlations with the adiposity categories as the outcomes. All statistical analyses were performed using SPSS version 21 (SPSS, Inc. Chicago, IL).

## Results

Out of 120 participants, 3 had missing accelerometer data (> 2 h of missing data over the three day collection period) and were excluded from the analysis. Therefore, 117 were included in the analytical sample (60 women, 57 men). Table 1 summarises the characteristics of the study population. Participants were aged 24–60 years (44 ± 9.2). Participants in different adiposity categories were of similar age. For example, normal BMI; 45 ± 8.4 years, overweight BMI; 38 ± 9.5 years; obese BMI; 49 ± 8.7 years. The percentage of smokers (16%) was similar to the UK population average (17%) [29] and participants who reported drinking alcohol weekly (47%) was slightly lower than the UK population average (58%) [30]. The percentage of participants (79%) who had a degree as their highest qualification was higher than the UK population average (27.2%) [31].

**Table 1:** Characteristics of the study population

Characteristics	
Sex (%)	
Male	51 (*n* = 60)
Female	49 (*n* = 57)
Age (years)^a^	44 ± 9
Height (cm)^a^	177 ± 5
Weight (kg)^a^	94 ± 8
Smoking status (%)	
Yes	16 (*n* = 19)
No	84 (*n* = 98)
Alcohol (%)	
Less than once per week	53 (*n* = 62)
1 or 2 times a week	38 (*n* = 44)
Several times a week or daily	9 (*n* = 11)
Education (%)	
University degree	79 (*n* = 92)
Postgraduate	21 (*n* = 25)

^a^ Numbers are in mean ± SD unless otherwise stated

Table 2 shows the Spearman correlations of accelerometer and questionnaire-assessed sedentary time, walking and MVPA in participants prior to splitting for weight status, stratified by adiposity group (BMI, BIA and WHR) and by sociodemographic group (smoking status, alcohol consumption and education). Modest to high correlations were observed for sedentary time (Rs = 0.49, 95%CI: 0.45, 0.53), walking (Rs = 0.58, 95%CI: 0.54, 0.61) and MVPA (Rs = 0.56, 95%CI: 0.53, 0.58). For sedentary time, modest correlations were observed across BMI, BIA and WHR adiposity groups (Rs = 0.22, 95%CI: 0.18, 0.25 to Rs = 0.38, 95%CI: 0.33, 0.40) and across sociodemographic groups (Rs = 0.21, 95%CI: 0.19, 0.24 to Rs = 0.32, 95%CI: 0.30, 0.34). Whereas, stronger correlations were observed for walking and MVPA, in participants with normal BMI and normal WHR (Rs = 0.43, 95%CI: 0.35, 0.49 to Rs = 0.58, 95%CI: 0.42, 0.64) overweight BMI and WHR (Rs = 0.30, 95%CI: 0.26, 0.35to Rs = 0.48, 95%CI: 0.44, 0.53) but not obese BMI and WHR (Rs = 0.24, 95%CI: 0.18, 0.32 to Rs = 0.48, 95%CI: 0.26, 0.42) or any BIA body fat groups (Rs = 0.14, 95%CI: 0.08, 0.22to Rs = 0.33, 95%CI: 0.25, 0.39). Modest correlations were also observed across sociodemographic groups for walking and MVPA (Rs = 0.21, 95%CI: 0.19, 0.24 to Rs = 0.36, 95%CI: 0.33, 0.35).

**Table 2:** Spearman correlation (rho) between questionnaire-assessed and accelerometer- assessed sedentary time, walking and MVPA according to adiposity group and sociodemographic characteristics

		Sedentary		Walking		MVPA	
		rho	95% CI	rho	95% CI	rho	95% CI
All participants		0.49	0.45, 0.53	0.58	0.54, 061.	0.56	0.53, 0.58
*Adiposity group*							
BMI category							
Normal weight ≤ 24.9	*n* = 37	0.36	0.30, 0.43	0.46	0.40, 0.49	0.43	0.35, 0.49
Overweight 25–29.9	*n* = 37	0.33	0.31, 0.36	0.33	0.29, 0.38	0.30	0.26, 0.35
Obese ≥30	*n* = 43	0.24	0.20, 0.30	0.34	0.26, 0.42	0.28	0.22, 0.36
BIA body fat %							
Average 14–20	*n* = 43	0.30	0.26, 0.34	0.31	0.27, 0.39	0.33	0.25, 0.39
High 21–25	*n* = 33	0.28	0.24, 0.31	0.32	0.28, 0.37	0.21	0.17, 0.26
Obese > 25	*n* = 41	0.22	0.18, 0.25	0.25	0.19, 0.32	0.14	0.08, 0.22
Waist-to-hip ratio							
Normal < 0.90	*n* = 43	0.38	0.33, 0.40	0.58	0.42, 0.64	0.46	0.38, 0.52
Overweight 0.90–0.99	*n* = 32	0.34	0.32, 0.37	0.48	0.44, 0.53	0.35	0.31, 0.40
Obesity > 1.00	*n* = 42	0.23	0.18, 0.25	0.33	0.25, 0.41	0.24	0.18, 0.32
*Sociodemographic Measures*							
Smoking status							
Yes	*n* = 19	0.26	0.21, 0.29	0.30	0.24, 0.36	0.28	0.20, 0.34
No	*n* = 98	0.31	0.29, 0.33	0.34	0.28, 0.39	0.26	0.22, 0.31
Alcohol							
Less than once per week	*n* = 62						
1 or 2 times a week or less	*n* = 44	0.29	0.23, 0.32	0.31	0.27, 0.35	0.30	0.26, 0.36
Several times a week or daily	*n* = 11	0.30	0.29, 0.33	0.32	0.29, 0.35	0.26	0.22, 0.31
GCSE/O level	*n* = 43	N/A		N/A		N/A	
A level	*n* = 32	N/A		N/A		N/A	
University degree	*n* = 43	0.32	0.30, 0.34	0.31	0.27, 0.35	0.32	0.24, 0.38
Postgraduate	*n* = 32	0.21	0.19, 0.24	0.36	0.33, 0.35	0.34	0.30, 0.39

Abbreviations: CI, confidence interval

The method of adiposity grouping used (BMI, BIA or WHR) influenced differences between accelerometer and questionnaire-assessed physical activity. Accelerometer and questionnaire-assessed measures were significantly different in participants categorised using BMI and BIA whereas several WHR groups did not reach statistical significance. For example, in obese participants, self-reported sedentary time was underreported by -195mins/day (*P* < 0.001) by -142mins/day (P < 0.001) and by -133mins/day (*P* < 0.001) when classified using BMI, BIA and WHR respectively. Self-reported walking in obese participants was over-reported by 19mins/day (*P* < 0.001), by 14mins/day (P < 0.001) and 5mins/day (*P* = 0.057) when classified using BMI, BIA and WHR respectively. Self-reported MVPA in obese participants was over-reported by 25mins/day (P < 0.001), by 24mins/day (P < 0.001) and 12mins/day (*P* = 0.052) when classified using BMI, BIA and WHR respectively. Similar trends were evident for normal and overweight participants (Table 3).

**Table 3:** Mean ± SD for questionnaire and accelerometer assessed sedentary time, walking and MVPA and their differences

		Sedentary (mins/day)				Walking (mins/day)				MVPA (mins/day)			
		Questionnaire	Acc^a^	Mean difference (Limits of agreement)	p	Questionnaire	Acc	Mean difference (Limits of agreement)	p	Questionnaire	Acc	Mean difference (Limits of agreement)	p
BMI	Normal	476 ± 84	597 ± 67	− 121 (− 227 to 35)	< 0.001	56 ± 29	42 ± 27	14 (−53 to 61)	< 0.001	125 ± 23	103 ± 19	21 (−33 to 56)	< 0.001
	Overweight	545 ± 91	704 ± 73	− 159 (− 345 to 27)	< 0.001	95 ± 27	77 ± 21	18 (−45 to 61)	< 0.001	121 ± 27	100 ± 21	20 (−43 to 63)	< 0.001
	Obese	628 ± 83	823 ± 94	−195 (− 394 to 5)	< 0.001	96 ± 23	77 ± 19	19 (−36 to 54)	< 0.001	70 ± 29	44 ± 27	25 (−52 to 63)	< 0.001
BIA	Average	422 ± 78	521 ± 86	−99 (− 283 to 85)	< 0.001	102 ± 27	83 ± 25	19 (−43 to 62)	< 0.001	133 ± 27	121 ± 25	19 (−41 to 65)	< 0.001
	High	532 ± 69	638 ± 98	− 106 (− 325 to 114)	< 0.001	78 ± 32	61 ± 28	17 (−56 to 70)	< 0.001	111 ± 32	82 ± 28	29 (−54 to 72)	< 0.001
	Obese	656 ± 73	798 ± 102	−142 (− 388 to 104)	< 0.001	43 ± 36	37 ± 29	14 (−67 to 74)	< 0.001	48 ± 36	24 ± 29	24 (− 66 to 75)	< 0.001
WHR	Normal	457 ± 82	560 ± 60	− 103 (− 251 to 46)	< 0.001	130 ± 22	123 ± 17	7 (−31 to 54)	**0.067**	169 ± 22	158 ± 17	11 (−27 to 58)	**0.058**
	Overweight	601 ± 78	644 ± 72	−43 (− 207 to 121)	< 0.001	78 ± 24	71 ± 21	7 (−40 to 54)	**0.063**	101 ± 24	92 ± 21	9 (−38 to 56)	**0.064**
	Obesity	712 ± 82	845 ± 97	−133 (− 307 to 41)	< 0.001	55 ± 25	50 ± 28	5 (−45 to 55)	**0.057**	82 ± 25	65 ± 28	12 (−43 to 56)	**0.052**

^a^ Abbreviations: Acc, accelerometer. Limits of Agreement expressed as the mean difference between methods ±1.96SD

Non-significant *P* values are shown in bold

The associations between adiposity group and accelerometer data were stronger than those between adiposity group and questionnaire data. Table 4 shows Spearman correlations for accelerometer and questionnaire-assessed physical activity related to BMI, BIA and WHR adiposity groups. Accelerometer-assessed physical activity showed modest to strong correlations. For example, correlations according to adiposity group for sedentary time were; 0.49 (BMI), 0.44 (BIA) and 0.53 (WHR), for walking were; 0.58 (BMI), 0.57 (BIA) and 0.70 (WHR), and for MVPA were; 0.54 (BMI), 0.48 (BIA) and 0.67 (WHR). However, questionnaire data showed only modest correlations with adiposity groups (0.24 to 0.37) regardless of which physical activity measure was considered (sedentary behaviour, walking or MVPA).

**Table 4:** Spearman’s rank correlations for questionnaire and accelerometer-assessed sedentary time, walking and MVPA with three adiposity categories

	Sedentary		Walking		MVPA	
	Questionnaire	Acc^a^	Questionnaire	Acc	Questionnaire	Acc
BMI	0.31	0.49	0.33	0.58	0.28	0.54
BIA	0.24	0.44	0.27	0.57	0.29	0.48
WHR	0.24	0.53	0.37	0.70	0.30	0.67

^a^ Abbreviations: Acc, accelerometer

## Discussion

Technological advances in the objective monitoring of physical activity now make it possible to obtain measures of sedentary behaviour, physical activity intensity and physical activity type from a single, body-worn accelerometer. In this study of normal, overweight and obese adults, we found that; 1) relationships between raw accelerometer and questionnaire-assessed sedentary behaviour were modest across all adiposity groups but walking and MVPA showed stronger associations in normal and overweight groups; 2) the use of WHR instead of BMI and BIA resulted in stronger agreement between accelerometer and questionnaire data; 3) associations between adiposity groups and accelerometer data were stronger than associations between adiposity groups and questionnaire data.

This study is the first to obtain several measures of physical activity from raw acceleration data in normal, overweight and obese adults. Methods exist which allow compatibility of raw acceleration signals with output from older devices such as the Actigraph GT1M [32, 33]. Correlations between accelerometer and questionnaire-assessed physical activity (rho = 0.49 to rho = 0.58) were equivalent to the highest reported in similar studies (from rho = 0.09 to rho = 0.58) using traditional devices [34]. A key recommendation regarding the objective monitoring of physical activity is that data should be collected and saved as raw acceleration signals to allow the storage of large amounts of movement data [35] and facilitate future comparisons of data across studies regardless of which accelerometer is used [36]. However, current recommendations make no reference to the analysis of raw accelerometer data in overweight and obese populations.

Associations between accelerometer and questionnaire-assessed sedentary behaviour in the present study were similar to those reported previously [37, 38]. Sedentary behaviour is an independent risk factor for weight gain [39], meaning research investigating sedentary behaviour in overweight and obese individuals is of increasing importance. Therefore, sedentary behaviour should be explicitly quantified in research and not simply defined by a lack of physical activity [40]. We used a questionnaire which asked specifically about daily sedentary behaviour and an accelerometer analysis which classified sedentary behaviour separately from other activity types [13]. Many accelerometers compress the raw acceleration signal into units called accelerometer counts. Accelerometer counts are generated when acceleration stays above a threshold value for a user defined epoch [41]. This renders some devices unable to differentiate between sedentary behaviours and short-duration, low intensity activities or the removal of the device [42]. Therefore, we opted to identify sedentary behaviour using activity type classification. Activity type classification involves the recognition of signature patterns in the raw acceleration signal which match activity types known by the algorithm [24]. This approach requires large amounts of computing power, hence it has only recently become feasible for use on modern desktop computers in studies involving large numbers of participants [43]. However, the classification analysis we used does not differentiate sleep from sedentary behaviour. Therefore, in the present study, participants kept a sleep diary - noting the times they went to sleep and when they woke up. It is likely the weaker associations observed for sedentary time compared to walking and MVPA is potentially due to the use of self-reported sleep duration. Nevertheless, sleep detection algorithms have recently become available for use with raw acceleration data [44, 45] and are incorporated into open-access analytical methods which also monitor MVPA [46]. Therefore, the use of accelerometer-based sleep detection analysis in future work would likely facilitate more accurate measurements of sedentary time. Nevertheless, our activity type analysis has been implemented in sedentary/slow moving populations to identify sedentary time and walking from raw acceleration data to good effect [47].

Walking is the most common type of physical activity, estimated to make up roughly one third of an adult’s daily physical activity [48, 49] and is therefore, commonly targeted by weight loss interventions [50–52]. Objective measurement methods such as pedometers [53] and some accelerometers [54] are deemed unsuitable for overweight and obese populations and likely contribute to the weak associations with questionnaire-assessed walking reported in previous studies [55–57]. However, as was the case when measuring sedentary behaviour, we used activity classification analysis [13] to detect walking. We found stronger associations between accelerometer and questionnaire-assessed walking in overweight and obese populations than previously reported in the literature [58, 59]. Accelerometer and questionnaire-assessed walking in participants with normal and overweight WHR differed by 7mins per day and in those with an obese WHR differed by just 5mins per day. Walking is thought to be detected more accurately in people who are more active [60]. However, obese participants were less active than their leaner counterparts. Therefore the high concordance between accelerometer and questionnaire-assessed walking is likely due to the use of activity classification techniques.

Due to the wealth of evidence associating MVPA with the greatest health benefits, most epidemiological studies assess physical activity expressed in MVPA (mins/day) [61]. We found stronger associations between accelerometer and questionnaire-assessed MVPA than previously reported in the literature. Accelerometer and questionnaire-assessed MVPA from the Whitehall II Study showed modest correlations (*r* = 0.33) [15]. However, authors used wrist-worn accelerometers, which are less burdensome to the participant, but provide a poorer measure of total body movement [42, 62]. Furthermore, care should be taken when monitoring MVPA in overweight and obese populations using accelerometers validated in non-obese adults. Since moderate (< 3–5.99 METs) and vigorous (> 6 METs) physical activity is based on MET cut-points derived from VO_2_ where 1MET = 3.5 mL/kg/min^− 1^, MVPA will be altered in overweight or obese populations since obesity is associated with reduced cardiorespiratory fitness [63] and diminished metabolic capacity [64]. Unsurprisingly, laboratory and free-living experiments suggest that accelerometers detect vigorous activity more accurately than lighter activity [65]. The cut point 70 mg, used on this study, has accurately classified MVPA previously [10]. However, this study involved adults with a normal body weight. Therefore, a more modest cut point may be more suitable for overweight and obese adults. Similarly, instead of using the IPAQ-S derived MET values to calculate MVPA [6], self-reported mins/day of moderate and vigorous physical activities were used for analysis. Collectively, these findings go some way in explaining why stronger associations between questionnaire and accelerometer-assessed MVPA were found in overweight and obese adults than previously reported.

Our study compared normal, overweight and obese adiposity groups. We found an overall trend where, irrespective of how adiposity group was classified (BMI, BIA or WHR), lower body weight resulted in stronger relationships between accelerometer and questionnaire-assessed sedentary behaviour, walking and MVPA (Rho = 0.14–0.58). Similarly, agreement decreased as body weight increased. Previously described studies typically compare accelerometer and questionnaire-assessed physical activity but have not compared normal with overweight/obese groups in the same study. Authors variously recommend questionnaires [66] or advocate the use of accelerometers [67]. This, combined with the use of varying measurement methods, makes direct comparisons with our study difficult. However, it is well established that higher body mass results in increased reporting bias [68]. This could potentially explain the reduction in agreement between accelerometer and questionnaire-assessed physical activity we observed in higher adiposity groups. Nevertheless, care should still be taken when comparing data from studies, where different methods of adiposity classification have been used.

The use of WHR instead of BMI and BIA resulted in stronger agreement between accelerometer and questionnaire-assessed sedentary behaviour, walking and MVPA compared to BMI and BIA. This could be due to the methodological strengths of WHR compared to BMI and BIA. By far the most popular marker of obesity used clinically [69] and in research [70] is BMI. Many studies have also used BIA to provide estimates of fat-mass and fat-free mass [71]. However, BMI is criticised for discounting actual body composition, namely fat-mass and fat-free mass [72] whilst BIA is criticised due to being altered by individual hydration levels [73] and when compared to the “gold standard” dual-energy X-ray absorptiometry, tends to underestimate fat-mass and overestimate fat-free mass [74]. Many studies encourage the use of WHR since it measures central fat unlike BMI and BIA. Studies have shown WHR may be a more informative way of classifying adiposity group (normal, overweight, obese) since this measurement focuses on abdominal or central fat. Excess central fat is linked to increased risk of metabolic syndrome [75], stroke [76], cardiovascular disease [77] and all-cause mortality [16]. Despite this, few studies have examined the effect of using different methods of adiposity classification on the relationship between questionnaire and accelerometer-assessed physical activity. We found significant differences between questionnaire and accelerometer-assessed sedentary time, walking and MVPA existed across BMI and BIA adiposity groups but less so for WHR. A previous study which compared physical activity assessed with questionnaire and wrist-worn raw accelerometer data using adiposity groups BMI, waist circumference, fat mass index (kg/m2) and BIA (although BIA was excluded from their analysis as some participants had renal insufficiency and thus, variable hydration levels) reported similar results [78]. Authors reported anthropometric measurements focusing on central adipose measurements were preferable to BMI when assessing physical activity using questionnaire and accelerometer in overweight and obese groups. Combined, these findings indicate WHR differentiated inactivity related excess adiposity most effectively and supports the use of anthropometric measures such as WHR, which estimate central fat, in studies monitoring physical activity and sedentary behaviour in overweight and obese individuals.

The associations between adiposity group and accelerometer data were stronger than those between adiposity group and questionnaire data. Unsurprisingly, these findings correspond with those from several other studies comparing subjective and objective measurement methods [79, 80]. A recent systematic review comparing accelerometry and questionnaire derived MVPA from both measurement methods advised the use of accelerometer data to obtain most complete physical activity information and reported similarly strong associations with accelerometer derived physical activity and adiposity group compared to questionnaire derived physical activity [81].

Our study is not without limitations. Firstly, although we tried to cover a large socioeconomic range, our participants had a higher than average education level compared to the general UK population. Physical activity in people from higher-socioeconomic groups is more accurately recalled [57, 82]. Secondly, our study does not extend to highest levels of adiposity and does not feature adults classed as extremely obese. Higher adiposity groups are important to reach since they are more difficult to treat [83]. Finally, the use of raw acceleration data in population based studies comes with large data processing and data storage needs. Although online platforms which use cloud technology are available more work is needed to make these applications widely accessible and user friendly to facilitate the use of raw acceleration signals in the measurement of physical activity and sedentary behaviour in overweight and obese populations.

## Conclusions

To our knowledge, this is the first study of its kind to compare physical activity and sedentary behaviour derived from raw accelerometer output with those from questionnaire in overweight and obese weight groups. Our findings assist researchers attempting to derive accurate, objective measures of physical activity and sedentary behaviour in overweight and obese adults and recommend the use of WHR. We show how to derive several measures of physical activity, namely sedentary behaviour, walking and MVPA, from a single, raw acceleration signal using open-source data analysis. Future studies can now work toward identifying the aspects of physical activity most important for health in overweight and obese populations by paying closer attention to measurement issues.

## Abbreviations

BIA: Bioelectrical impedance analysis
BMI: Body mass index
CI: Confidence interval
COPD: Chronic obstructive pulmonary disorder
ENMO: Euclidian Norm Minus One
GGIR: GENActiv and GENEA in R
IPAQ-S: International Physical Activity Questionnaire Short form
MVPA: Moderate-to-vigorous physical activity
NHANES: National Health and Nutritional Examination Survey
Rs: Spearman’s rank correlation
WHR: Waist-to-hip ratio
